# Phytochemical Characterization of Commercial Processed Blueberry, Blackberry, Blackcurrant, Cranberry, and Raspberry and Their Antioxidant Activity

**DOI:** 10.3390/antiox8110540

**Published:** 2019-11-10

**Authors:** Zoriţa Diaconeasa, Cristian I. Iuhas, Huseyin Ayvaz, Dumitriţa Rugină, Andreea Stanilă, Francisc Dulf, Andrea Bunea, Sonia Ancuța Socaci, Carmen Socaciu, Adela Pintea

**Affiliations:** 1 Faculty of Food Science and Technology, University of Agricultural Science and Veterinary Medicine Cluj-Napoca, Calea Mănăştur 3-5, 400372 Cluj-Napoca, Romania; zorita.sconta@usamvcluj.ro (Z.D.); andreea.stanila@usamvcluj.ro (A.S.); sonia.socaci@usamvcluj.ro (S.A.S.); carmen.socaciu@usamvcluj.ro (C.S.); 2 Faculty of Medicine, Iuliu Hatieganu University of Medicine and Pharmacy, 400372 Cluj-Napoca, Romania; iuhascristianioan@yahoo.co.uk; 3 Department of Food Engineering, Canakkale Onsekiz Mart University, 17020 Canakkale, Turkey; huseyinayvaz@comu.edu.tr; 4 Faculty of Veterinary Medicine, University of Agricultural Science and Veterinary Medicine Cluj-Napoca, Calea Mănăştur 3-5, 400372 Cluj-Napoca, Romania; apintea@usamvcluj.ro; 5 Faculty of Agriculture, University of Agricultural Science and Veterinary Medicine Cluj-Napoca, Calea Mănăştur 3-5, 400372 Cluj-Napoca, Romania; francisc.dulf@usamvcluj.ro; 6 Faculty of Animal Science and Biotechnology, University of Agricultural Science and Veterinary Medicine Cluj-Napoca, Calea Mănăştur 3-5, 400372 Cluj-Napoca, Romania; andrea.bunea@usamvcluj.ro

**Keywords:** anthocyanins, antioxidants, berry, jams, phenolics, volatiles

## Abstract

Being delicious and containing strong disease-fighting agents, berries represent an increasing proportion of fruits consumed nowadays in our diet. However, berries are highly perishable as fresh and, therefore, they are usually processed into various products to extend their shelf-life and availability throughout the year. Among the fruit-containing products, jam is one of the most common due to its nourishing properties, its low production costs, and its accessibility for a lengthy period. Rather than home preparation, consumers nowadays increasingly prefer to purchase commercial jams from markets due to its convenience. Although fresh berries have been extensively studied for their phenolic compounds, a limited number of studies investigating commercially manufactured jams have been conducted so far. Considering this, the objective of this study was to assess the total phenolic, flavonoid, and anthocyanin content and the antioxidant activity of five commonly consumed commercial berry jams (blueberry (*Vaccinium myrtillus*), blackberry (*Rubus fruticosus*) and blackcurrant (*Ribes nigrun*) mixture, blackcurrant (*Ribes nigrun*), cranberry (*Vaccinium macrocarpon*) and raspberry *(Rubus idaeus*)) collected from the market. Even though a possible loss of phenolics, anthocyanins, and a decrease of radical scavenging activity may occur during jam processing and subsequent storage, our data indicated that the selected commercial jams remained good sources of nutritive molecules with antioxidant properties based on the high levels of total phenolics, flavonoids, anthocyanins, and elevated antioxidant activities determined in this study. Additionally, the samples were characterized by GC-MS for their volatile profiles, and terpenes were found to be the dominating class covering more than 74% of volatile compounds in the jams.

## 1. Introduction

Reactive oxygen species are either naturally formed in our body as a result of human metabolic pathways or can be generated through various environmental and physiological processes. The resulting oxidative stress in our body has been associated with several chronic and degenerative diseases [1]. In fighting against these types of diseases, the importance of a proper diet, as opposed to the common use of synthetic medicines, has been recently well-recognized. Accordingly, nutrients mainly sourced from fruits and vegetables have been more intensively explored [2]. Nowadays, high consumption of fruits and vegetables through our diet, thanks to their abundance in antioxidants and ability to quench the reactive oxygen species formed in the human body, is encouraged worldwide [3]. Among the fruits consumed in our diet, delicious and strong disease-fighting berries entail an increasing percentage. Berries are known to contain high levels of phenolics, flavonoids, and anthocyanins and, therefore, exhibit a strong antioxidant capacity. Thus, there are many studies available in the literature investigating berries from a chemical point of view as in the fresh form [4,5,6]. However, being quite perishable, berries are commonly processed into various products to extend their availability and shelf-life [7,8]. They are widely used as ingredients in beverages, ice cream, yogurt, milkshakes, jams and jellies, smoothies, dried or canned products, as well as dietary supplements. Among these products, jams are one of the most popular due to their low-cost and all year accessibility [9,10]. Berry jams are usually made by combining similar ratios of berry fruits and sugar and further cooking the mixture. Among the other phytochemicals, anthocyanins from fruits contribute to the color, nutritive, and the health properties of the jam [11,12]. However, polyphenols, including flavonoids or anthocyanins, are not very stable in the food matrix and some degradation may occur during jam processing and following storage due to various factors, including temperature, pH, anthocyanin concentration and structure, oxygen, light, and enzymes. Their degradation during processing may cause some color loss in jams, influencing the consumers’ acceptance [13,14]. Therefore, proper temperature and time combinations in jam processing must be chosen to ensure anthocyanin stability and to preserve the antioxidant capacity [15]. Good quality jams are expected to present their nutritional and sensorial characteristics substantially throughout their extended storage [16]. 

To the best of our knowledge, literature studies on jams so far have mostly focused on the bioactive compounds and related properties in raw fruits, how they were altered upon processing, and throughout their storage [15,16,17,18]. It is also crucial to mention that conflicting results have been reported in these studies as to how anthocyanins, phenolics, and radical scavenging activities were changed upon jam processing and storage. Additionally, in all these studies, berries were processed into jams following the home-cooking methods. However, consumption of commercial jams has become more and more popular every day due to the modern lifestyles that consumers follow. We believe it is critical for the food industry that the commercial jam products they produce not only meet the consumer satisfaction for their taste, texture, and appearance, but also potential health benefits. Accordingly, the main reason that drove us towards this study was the greater consumption of commercial jams in comparison with home-made ones and the limited research available in the literature on the commercial jams sold in the market. Our goal was to analyze some of the most common commercial jams of purple-red berries (blueberry (*Vaccinium myrtillus*), blackberry (*Rubus fruticosus*) and blackcurrant (*Ribes nigrun*) mixture, blackcurrant (*Ribes nigrun*), cranberry (*Vaccinium macrocarpon*) and raspberry *(Rubus idaeus*)) for their contents of anthocyanins, total phenolics, total flavonoids, and to determine their antioxidant activity and volatile profiles to evaluate if these parameters of interest were still in satisfactory levels in commercial jams ready to be sold in market shelves. An additional goal of this study was to identify the volatile compounds and determine the individual relative concentration of each compound leading to the characteristic flavor of these selected commercial jams.

## 2. Materials and Methods 

### 2.1. Materials

HPLC-grade water, LC-MS-grade methanol (99.9%), formic acid (88%), ethyl acetate, hydrochloric acid (12N) 6-hydroxy-2,5,7,8-tetramethylchroman-2-carboxylic acid (Trolox) (98%), and 2,2-azinobis (3-ethylbenzothiazoline-6-sulfonic acid) diammonium salt (ABTS) (98%) were purchased from Sigma-Aldrich (Darmstadt, Germany). Standards of cyanidin (95%), and cyanidin-3-*O*-arabinoside (97%), cyanidin-3-*O*-glucoside (95%), and cyanidin-3-*O*-galactoside, pelargonidin-3-*O*-glucoside (90%) were acquired from Polyphenols Laboratories AS (Sandnes, Norway). Commercially available berry jams made of blueberry, blackberry-blackcurrant mixture, blackcurrant, cranberry, and raspberry were bought from a supermarket in Vienna, Austria. The ingredients listed on the label of the jams are shown in Table 1. All the jams, except the blackcurrant jam, contained 50 g fruits and additional sugar, citric acid, and pectin for 100 g jam. Blackcurrant jam contained 40 g blackcurrant fruits and no pectin in the preparation of 100 g jam. All used berries were obtained from organic agriculture. 

### 2.2. Extraction 

Each berry jam (three samples for each jam) was homogenized using an Ultraturax homogenizer (Ultra-Turrax, Model Miccra D-9 KT, Digitronic GmbH, Bergheim, Germany) and 5 g of it was used for the further analyses. Ten mL of acidified methanol (0.3 % HCl (*v/v*)) was added, and the mixture was shaken for 20 min on a magnetic stirrer in the dark. The supernatant was collected, and the extraction procedure was repeated until the samples were colorless. The collected extracts were evaporated at 35 °C under reduced pressure (Rotavapor R-124, Buchi, Switzerland), dissolved in acidified water, and filtered through 0.45 μm Millipore filter before HPLC and antioxidant analyses. To avoid any anthocyanin pigment loss during the experiment, all the procedures were carried out in subdued light and under controlled conditions. 

### 2.3. Phenolic Content 

Total phenolics were measured following the Folin–Ciocalteau colorimetric protocol from Singleton et al. [19] with some minor modifications. Briefly, aliquots (25 μL) of the extracts, a gallic acid calibration standard, and water blank were placed into separate plastic cuvettes. Then, 1.8 mL distilled water was added to each cuvette, followed by 120 μL of Folin–Ciocalteu reagent, thoroughly mixed and incubated for 5 min. After incubation, 340 μL of sodium carbonate (7.5% Na_2_CO_3_ in water) was added, mixed and allowed to incubate for 90 min at room temperature. Then, the absorbance of the samples, standards, and blanks was read at 750 nm using a microplate reader (BioTek Instruments, Winooski, VT, USA). The absorbance of the blank was subtracted from all readings, and a calibration curve was created using the standard. Total phenolic content was calculated as gallic acid equivalents (GAE) based on the gallic acid calibration curve. The calibration curve was plotted using gallic acid solutions at concentrations of 50–450 μg/mL (*R*^2^ = 0.9928). The analyses were performed in triplicate.

### 2.4. The Total Flavonoid Content

The total flavonoid content of the extracts was determined by a colorimetric method, as described previously in other studies [20,21]. One mL aliquot of the appropriately diluted extract was added to a 5 mL volumetric flask. At zero time, 300 μL of 5% NaNO_2_ was added to the flask. After 5 min incubation, the mixture was treated with 300 μL AlCl_3_ (10%) and incubated for 6 min. Then, 2 mL of 1 M NaOH was added. The solution was mixed well, and the absorbance at 720 nm versus water blank was recorded using a spectrophotometer (JASCO V-630 series, International Co., Ltd., Tokyo, Japan). The flavonoid content calculated using a quercetin standard curve and expressed as mg QE/100 g FW. The calibration curve was prepared by preparing quercetin solutions at concentrations of 5–50 μg/mL (*R*^2^ = 0.9932). Each determination was carried out in triplicate. 

### 2.5. 2ʹ-Azino-bis (3-ethlylbenzothiazoline-6-sulfonic acid) (ABTS) Radical Cation-Decolorization Assay 

Among the options available, ABTS assay was selected for measuring antioxidant content of berry jams since ABTS assays are known to be highly correlated with the phenolic content and oxygen radical absorbance capacity in addition to being a simple, fast, and cost-effective analysis. Further advantages of the ABTS method over other methods available can be found in the literature [22]. The scavenging ability of the extracts against radical anion ABTS was determined according to the procedure described with a modification to be adapted to 96-well microplates [23]. Blue-green color ABTS˙^+^ solution was obtained by mixing 5 mL of 7 mM ABTS˙^+^ and 80 µL of 2.45 mM potassium persulfate. The reaction was carried out in the dark at room temperature for 16 h before its use. ABTS˙^+^ working solution was obtained by diluting the stock solution with ethanol, in order to obtain an absorbance value of 0.70 ± 0.02 AU at 734 nm. Once the working solution was prepared, 20 µL Trolox at different concentrations or fruit extract were added to 170 µL ABTS˙+ solutions, and the absorbance was measured at 734 nm after 6 min of incubation in the dark at room temperature using a microplate reader. The results were calculated according to the standard curve of Trolox (0–400 µM, *R*^2^ = 0.997) and expressed as μmol Trolox equivalents (TE)/g FW.

### 2.6. HPLC-PDA-MS Identification and Quantification of Anthocyanins

HPLC analysis was performed on an Agilent 1200 system (Chelmsford, MA, USA) equipped with a binary pump delivery system LC-20 AT (Prominence), degasser DGU-20 A3 (Prominence), and diode array SPD-M20 A UV–VIS detector (DAD). The separation was achieved on a Luna Phenomenex C_18_ column (5 µm, 25 cm × 4.6 mm) and the column temperature was maintained at 25 °C. The mobile phases were 4.5% formic acid in bidistilled water (solvent A) and acetonitrile (100%) (solvent B) with a solvent flow rate set at 0.5 mL/min. The gradient elution system started with 10% B for 9 min. The percent of B increased to 12% at 17 min and continued up to 25% B at the 20th min. Between the 20th and 55th min, the percentage of B was 90%. The absorbance was monitored at 520 nm. The compound identification and peak assignments were done based on their retention times, UV–VIS spectra as well as comparisons to standards and published data. As a confirmation, the samples were analyzed by HPLC-MS as well. The mass spectrometric data were obtained using a single quadrupole 6110 mass spectrometers (Agilent Technologies, Chelmsford, MA, USA) equipped with an ESI+ probe. The measurements were performed in the positive mode with an ion spray voltage of 3000 V, and a capillary temperature of 350 °C. Data were collected in full scan mode within the range of 280–1000 *m/z*. The quantification of anthocyanins was done by using cyanidin 3-galactoside as standard. The identification of anthocyanins was carried out based on the molecular mass determination (*m/z* values), main fragments, the elution order, and comparison with the literature data. 

### 2.7. Volatile Profile by ITEX/GC-MS

The extraction of volatile compounds was performed using the in-tube extraction technique (ITEX) as described in our previous work using 5 g of the sample [24]. The analysis of volatile compounds was carried out on a GC MS QP-2010 (Shimadzu Scientific Instruments, Kyoto, Japan) model gas chromatograph-mass spectrometer. The volatile compounds were separated on a Zebron ZB-5ms capillary column of 30 m × 0.25 mm and 0.25 μm film thickness. The carrier gas was helium with a flow rate of 1 mL/min and the split ratio of 1:5. Oven settings started at 35 °C (hold for 10 min) to 105 °C, at 5 °C/min increments. Then, the temperature was increased by 2 °C/min until 135 °C and by 25 °C/min up to 230 °C, where it was held for about 5 min. The injector, ion-source, and interface temperatures were set at 250 °C. The MS detection was used for the qualitative analysis on a quadrupole mass spectrometer operating in full scan (40–350 *m/z*) electron impact (EI) at 70 eV ionization energy. The volatile compounds were tentatively identified by first comparing the mass spectrometric information of each chromatographic peak to NIST27 and NIST147 mass spectra libraries (considering a minimum similarity of 85%) and then whenever possible by comparison with retention indices drawn from www.pherobase.com or www.flavornet.org (for columns with a similar stationary phase to the ZB-5ms column). This technique offers a qualitative assessment of volatile compounds, so the relative percentage of each compound was estimated as a fraction of its integrated ion area from total ion chromatograms (TIC) area (100%). 

### 2.8. Statistical Analysis 

Statistical differences among samples were estimated using ANOVA (repeated measures ANOVA; Tukey’s multiple comparison test; GraphPad Prism Version 7.0, Graph Pad Software Inc., San Diego, CA, USA). A value of *p* < 0.05 was considered statistically significant.

## 3. Results

### 3.1. Phenolic Content 

Randomly-selected five berry jams (blueberry, blackberry and blackcurrant mixture, blackcurrant, cranberry, and raspberry) were analyzed for their total phenolic content using Folin–Ciocalteu assay. Based on the results shown in Table 2, statistically significant differences among the total phenolic contents of the jams were recorded. The content of total phenolics ranged between 170.32 and 473.91 mg of GAE/100 g FW, agreeing with the literature range of 280–450 mg GAE/100 g FW as reported by Šavikin et al. [25] on bilberry, black raspberry, and blackcurrant jams. The blackcurrant jam contained the highest total phenolic content (473.91 mg GAE/100 g FW), whereas raspberry jam demonstrated to have the lowest phenolic content (170.32 mg GAE/100 g FW). Blackcurrant jam appears to have the highest phenolic content, and a mixture of blackberry (70%) and blackcurrant (30%) prepared as jam contained remarkably less phenolics (260.74 mg GAE/100 g FW). However, a jam prepared only with blueberries has 23% more phenolic compounds than the blackberry (70%) and blackcurrant (30%) mixture. 

It is known that a remarkable reduction in polyphenol content of the fruits occurs as a result of heat treatment during jam production. The same authors evaluated the effect of jam processing and storage on blackcurrant and black raspberry jams and reported that phenolic content decreased during both processing and storage (up to 80%) [25]. They determined the total phenolic content of fresh berries ranging from 380 (blackcurrant) to 1660 (black raspberry) mg GAE/100 g FW, while in their corresponding jams, the levels were only 280 (blackcurrant) and 290 (black raspberry) mg GAE/100 g FW [26]. In another study, a reduction in total polyphenols of strawberry jams due to heat treatment was observed, and this loss was attributed to the enzymatic and auto-oxidation of the compounds and the breakdown of anthocyanins or flavonoids caused by high temperatures [16]. Upon further comparison, Šavikin et al. [25] found that fresh blackcurrants contained 380 mg GAE/100g FW and the level was reduced to 280 mg GAE/100g FW when processed into blackcurrant jam. At the end of 9 month storage at room temperature, the authors reported the phenolic content of blackcurrant jam to reach back to 660 mg GAE/100g FW. Similarly, Pineli et al. [15] investigated the changes in phenolic content of strawberry jams during 120 days and reported the values to be 235 mg GAE/100g FW on zero-day storage and 236 at 120 days of storage. Yeon Seo et al. [27] also measured the total phenolic content of the jams they produced and reported about 153 and 72 mg GAE/100g FW total phenolics for blueberry and black raspberry mixture jam and raspberry and strawberry mixture jam, respectively. Another study reported total phenolics of 495 mg GAE/100g FW in frozen bilberries and the level decreased to as low as 142 mg GAE/100g FW after processing into jam and eight months of storage [28]. Although berry types used in this study and the relevant studies compared in literature may differ, the total phenolic contents of our commercial jams were reasonably similar to processed and stored samples mentioned in the previous studies. 

### 3.2. Total Flavonoid Content

The total flavonoid contents of the samples were evaluated by using the aluminum chloride colorimetric method, and the results are shown in Table 2. The levels of the total flavonoids ranged between 2.61 mg QE/100 g FW in the blueberry jam and 11.43 mg QE/100 g FW in blackcurrant jam. Similar to the total phenolic content, blackcurrant jam contained the highest total flavonoid concentration. Additionally, in the case of blackberry and blackcurrant mixture, the total flavonoid content was again significantly lowered (7.26 mg QE/100g FW) compared to that of blackcurrant jam (11.43 mg QE/100 g FW). A recent study reported a flavonoid content of 9.5 mg QE/100 g FW for a mixed raspberry and strawberry jam, while the flavonoid content was 6.6 times higher for mixed blueberry and Korean black raspberry jam [27]. Their quantitative differences to our results could be due to different cultivars used for jam preparation, as well as the different harvesting time, the manufacturing procedure, or even the storage conditions used. 

### 3.3. Antioxidant Potential

The antioxidant potential of the samples was measured by ABTS assay and found to be ranging between 6.10 and 36.56 μM Trolox/g FW with the highest antioxidant activity being observed for blackcurrant sample. ABTS assay is a colorimetric method which can be applied to samples containing hydrophilic, lipophilic, and highly pigmented antioxidant compounds [28,29,30,31].

Based on the data values obtained one can observe that regarding the total phenolics, total flavonoid content and their antioxidant activity we have blackcurrant > cranberry > blackberry and blackcurrant > raspberry > blueberry. Regarding the correlation between analyzed compounds and antioxidant activity we have a strong correlation between the total flavonoid and antioxidant activity, *r* = 0.95. Our values for scavenging activity were similar to those reported by Bunea et al. [29], who analyzed seven different blueberry cultivars and reported their antioxidant activities determined by ABTS assay as ranging from 6.05 to 11.96 μM Trolox/g FW. A recent study reported that after exposure to several heat treatments, the total anthocyanin content in the blueberry jam was lower than that of fresh blueberries [17]. There are others studies which sustain that the cooking procedure lowers the antioxidant level and, also, the addition of ingredients such as sugar are able to dilute the final concentration of the antioxidants, but products made from berries were still rich sources of antioxidants [30]. 

### 3.4. HPLC-PDA-MS Identification and Quantification of Anthocyanins

**Figure 1:**
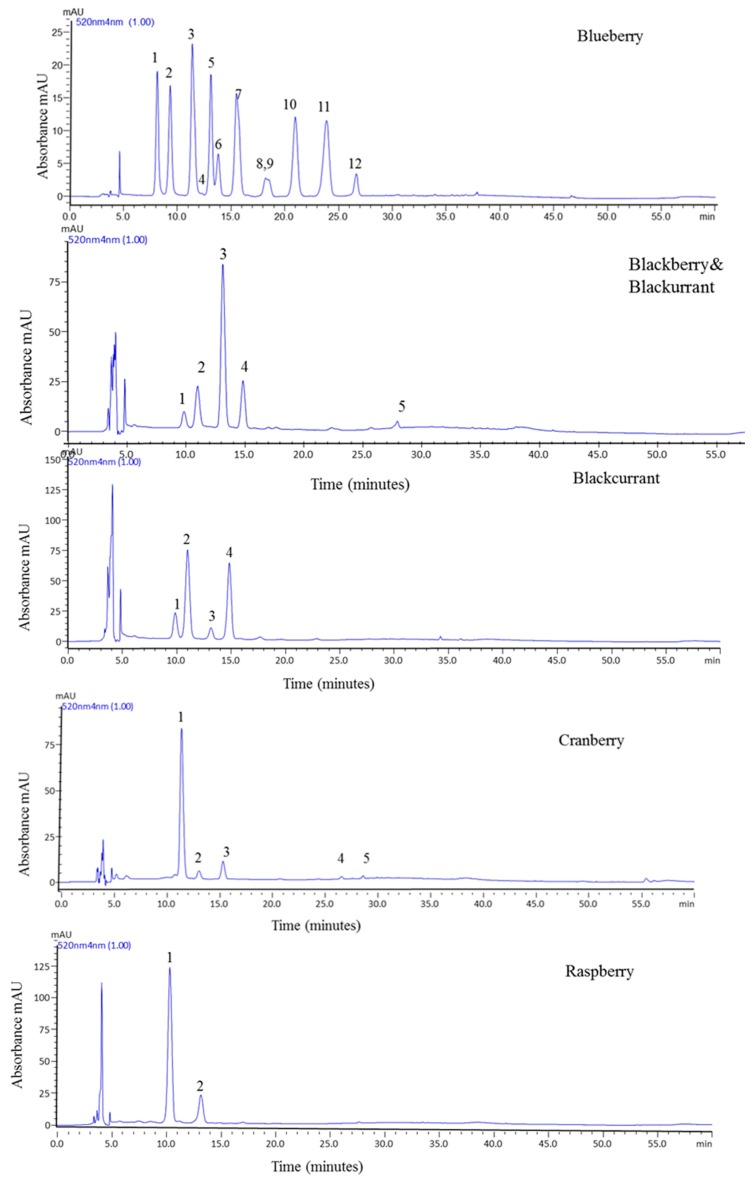
HPLC-PDA chromatograms of jam samples recorded at 520 nm.

HPLC-PDA-MS identification of anthocyanins was made based on their retention time, UV–VIS spectra and mass spectral analysis compared with standards and literature data. The representative HPLC chromatograms with detection at 520 nm wavelength are displayed in Figure 1. Among the samples, the blueberry profile contained the most complex anthocyanin pattern with 12 different anthocyanins identified Tentative identification and quantification of anthocyanins are presented in Table 3. The specific retention times (t_R_), UV–VIS maximum absorption wavelengths, m/z values, main fragment ion mass, and concentration of each anthocyanin are also given in Table 3. Anthocyanin content was expressed as mg cy-3-*O*-gal/100 g FW. The highest anthocyanin concentration was found to be in cranberry (17.13 mg /100 g FW) and blackcurrant (16.17 mg/100 g FW) jam samples while the lowest was in raspberry jam (10.89 mg /100 g FW). We found anthocyanin levels of blueberry and blackberry-blackcurrant mixture in a jam to be 15.21 and 14.06 mg/100 g FW, respectively. These levels were comparable with Pineli et al. [16], who reported the total anthocyanin level in strawberry jams as 25.8 mg/100 g FW right after jam processing and significantly lower level of 13.5 mg/100 g FW after 120 days of storage at room temperature.

Our results were also comparable to the data reported by Yeon Seo et al. [27]. The authors reported a total anthocyanin level of 23.1 mg/100 g FW for a mixed raspberry and strawberry jam and 6.1 mg/100 g FW for mixed blueberry and Korean black raspberry jam. Additionally, Savikin et al. [26] evaluated the effect of jam processing and storage on blackcurrant and black raspberry jams and have reported that total anthocyanins (%) of fresh blackcurrant (0.16%) and black raspberry (0.37%) decreased an average of 0.05% and 0.17% after jam processing [25].

From the HPLC-PDA chromatograms and LC-MS data, the anthocyanins found in our samples were identified and quantified. Except for pelargonidin, all of the common aglycons found in nature (cyanidin, delphinidin, peonidin, petunidin, and malvidin) were identified in our samples with different substitutions. Among the 12 anthocyanins determined in blueberry jam extract, there were three delphinidin, three cyanidin, two petunidin, one peonidin, and three malvidin derivatives with -3-*O*-glucoside, -3-*O*-galactoside, and -3-*O*-arabinoside substitutions. The anthocyanins present in our blueberry jam were identical with those reported previously in fresh blueberries [29,31,32].

Although the amounts of delphinidin-3-*O*-galactoside, delphinidin-3-*O*-glucoside, cyanidin-3-*O*-galactoside, and petunidin-3-*O*-galactoside were significantly higher compared with the other identified compounds, none of the individual anthocyanins seemed to be dominant in blueberry jam. Among the four anthocyanins identified in blackcurrant jam, delphinidin-3-O-rutinoside (7.89 mg/100 g FW) and cyanidin-3-*O*-rutinoside (4.74 mg/100 g FW) were present at much higher levels than the other two anthocyanins. The values calculated for the other two anthocyanins were 2.75 mg/100 g for delphinidin-3-*O*-glucoside and 0.78 mg/100 g for cyanidin-3-*O*-glucoside. These results were consistent with the data reported on fresh blackcurrant samples in the literature [33]. In the mixed jam containing 70% blackberry and 30% blackcurrant, two delphinidin and three cyanidin derivatives were determined. Cyanidin-3-*O*-malonyl-glucoside was the most abundant (9.189 mg/100 g FW out of 14.06 mg/100 g FW). As expected, this jam contained the same four anthocyanins found in blackcurrant jam (delphinidin-3-*O*-glucoside, delphinidin-3-*O*-rutinoside, cyanidin-3-*O*-glucoside, and cyanidin-3-*O*-rutinoside). These four anthocyanins were also present in fresh blackberry samples according to literature data. Here, an additional acylated anthocyanin, cyanidin-3-*O*-malonyl-glucoside was identified, which is characteristic to blackberries [25,33,34]. Cranberry jam, which is a slightly sour-tasting jam served as a garnish to meat in Austria, contained three cyanidin and two malvidin derivatives, with cyanidin-3-*O*-galactoside representing about 82%.

Raspberry jam had only two cyanidin derivatives (cyanidin-3-*O*-sophoroside and cyanidin-3-*O*-sophoroside-5-rhamnoside), the data being in accordance with previous studies [31,35]. Garcia-Viguera et al. investigated the anthocyanin profile of Zeva and Heritage raspberry cultivars [36]. They reported four cyanidin derivatives (-3-sophoroside, 3-glucosylrutinoside, 3-glucoside, and 3-rutinoside) in Zeva and only 3-*O*-sophoroside and -3-*O*-glucoside in Heritage with cyanidin-3-*O*-sophoriside being much more abundant in Heritage. Upon the comparison with the literature data on raspberry jam anthocyanins, our findings indicate that the raspberry cultivar, used to prepare the commercial jam, was most likely *Rubus ideaeus* var. Heritage. A significant reduction on the total anthocyanin content per gram of fresh fruit during jam processing for the cultivar Heritage was also reported previously [36], suggesting that thermal processing induces a loss of about 27% in anthocyanins of purees, and this loss can further accelerate during storage [37].

### 3.5. The Volatile Profile by ITEX/GC-MS

**Figure 2:**
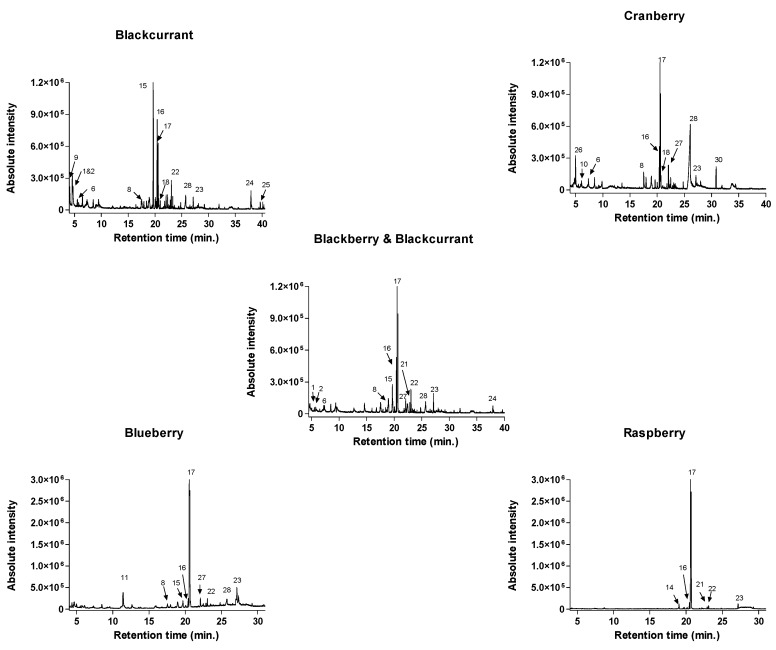
Chromatograms (TIC, total ion chromatogram) of headspace ITEX/GC–MS analysis of volatiles from the selected jams. The numbering of the peaks refers to Table 3.

For consumers, the flavor is a significant factor in the decision-making process when buying a specific food product. In addition to the tastants (e.g., organic acids and sugars), the volatile aromatic compounds have an essential role in the development of food flavor [38]. A summary of aroma active volatile from analyzed jams by ITEX/GC-MS technique is shown in Table 3. A total of 30 compounds were separated, of which 28 were identified, including five alcohols, three aldehydes, four esters, and 13 terpenoids. Based on the relative concentration of volatiles, terpenes (terpenoids) were the most abundant class (in terms of total chromatographic area), covering over 74% of the volatile profile of the overall jams (Figure 2). In the case of cranberry, blueberry, raspberry, and blackberry/blackcurrant samples, limonene was the primary terpene while for the blackcurrant sample, three dominant terpenes were identified: 3-carene (28.59%), *p*-cymene (14.96%), and limonene (11.40%), all imparting a fruity, citrus-like aroma. 

Terpenes, such as linalool, contributed with “sweet,” “floral,” “fruity,” and “citrus” aroma notes and α- terpineol contributed with “woody” and “piney” aroma notes (Table 4). Aldehydes, including hexanal and heptanal, are the next most abundant group in jams contributing to the “fresh green” and “fruity” aroma notes. Blackcurrant jam is the most abundant jam in aldehydes (3.35%). The esters mainly impart fruity and floral attributes to foods. The primary esters identified in the jam samples were methyl butanoate (in blackcurrant jam) and ethyl 3-methylbutanoate (in the blueberry jam). Ethyl 3-methylbutanoate has been previously reported in lowbush blueberries [39]. Alcohols, including 3-methyl 1-butanol, 2-methyl 1-butanol, furfuryl alcohol, 1-hexanol, and 2-heptanol, were identified in the jam samples (Table 4). Earlier reports confirmed that 2-heptanol contributed a “musty” aroma to southern highbush blueberries [39]. 

## 4. Conclusions

In this present work, randomly-selected commercial berry jams from the market were investigated for their phenolic and antioxidant properties to elucidate whether commercial jams are still good sources of these health-promoting properties. Based on a comparison with literature data on fresh berries and home-made jams, we determined that comparable levels of polyphenolic quality parameters can still be found in the commercial jams ready to be sold in the markets. Therefore, our data sustain this argument and prove that the analyzed jams in this study (blueberry, blackberry, blackcurrant, cranberry, and raspberry) could be considered rich sources of nutritional substances with high antioxidant potential. Furthermore, the headspace ITEX/GC–MS technique successfully applied for the extraction and analysis of volatile compounds from selected jams helped us to identify 28 volatiles responsible for the aroma of the jams, including alcohols, aldehydes, esters, and terpenes, with terpenes being the most abundant.

## Figures and Tables

**Table 1:** Ingredients listed on the label of each berry jam used in this study.

Jam	Ingredients for 100 g Final Product
Blueberry	50 g blueberry, sugar, citric acid, pectin
Blackberry and Blackcurrant	50 g berry (blackberry 70%, blackcurrant 30%), sugar, citric acid, pectin
Blackcurrant	40 g blackcurrant, sugar, water, citric acid
Cranberry	50 g cranberry, sugar, citric acid, pectin
Raspberry	50 g raspberry, sugar, citric acid, pectin

**Table 2:** Total phenolics, flavonoids, and antioxidant activity of the five berry jams.

Jam Type	Total Phenolics GAE mg/100g FW	Total Flavonoid mg QE/100g FW	Antioxidant Activity (μM Trolox/g FW)
Blueberry	360.44 ^b^	2.61 ^e^	6.10 ^d^
Blackberry & Blackcurrant	260.74 ^d^	7.26 ^c^	18.26 ^b^
Blackcurrant	473.9 1^a^	11.43 ^a^	36.56 ^a^
Cranberry	310.40 ^c^	9.46 ^b^	20.21 ^b^
Raspberry	170.32 ^e^	4.43 ^d^	10.10 ^c^

Mean values of triplicate determinations expressed on a fresh weight basis. The different letters (a–e) within the same column represents are significant differences between mean values (*p* < 0.05). For abbreviations see Section 2 (Materials and Methods). GAE, QE, FW.

**Table 3:** Retention times, UV–VIS max. absorption wavelengths, tentative identification, and concentration of anthocyanins in berry jams expressed in mg cyanidin-3-*O*-galactoside per gram of jam (fresh weight).

Peak No	t_R_ (min)	UV–VIS λ_max_	Molecular Ion m/z	Fragment Ion m/z	Tentative Identification	Concentratin (mg cy-3-O-gal/100 g FW)
**Blueberry**						
1	8.1	276,526	465	303	Delphinidin-3-O-galactoside	1.866 ^a^
2	9.3	276,524	465	303	Delphinidin-3-O-glucoside	1.911 ^a^
3	11.3	279,517	449	286	Cyanidin-3-O-galactoside	1.821 ^a^
4	12.23	276,524	435	303	Delphinidin-3-O-arabinoside	0.964 ^e^
5	13.1	280,516	449	287	Cyanidin-3-O-glucoside	1.395 ^b^
6	13.7	276,526	479	317	Petunidin-3-O-galactoside	1.899 ^a^
7	15.4	279,517	419	287	Cyanidin-3-O-arabinoside	1.189 ^d^
8	18.2	276,526	463	301	Peonidin-3-O-galactoside	0.587 ^g^
9	18.4	276,526	449	317	Petunidin-3-O-arabinoside	0.663 ^f g^
10	20.9	276,527	493	331	Malvidin-3-O-galactoside	1.183 ^d^
11	23.8	276,526	493	331	Malvidin-3-O-glucoside	1.217 ^c d^
12	26.6	276,528	465	331	Malvidin-3-O-arabinoside	0.524 ^g^
15.219						
**Blackberry & Blackcurrant**						
1	9.80	275,523	465	303	Delphinidin-3-O-glucoside	0.616 ^c^
2	10.95	272,526	300	283/252	Delphinidin-3-O-rutinoside	2.314 ^b^
3	13.09	279,516	535	287	Cyanidin-3-O-malonyl-glucoside	9.189 ^a^
4	14.81	278,519	449	287	Cyanidin-3-O-glucoside	2.259 ^b^
5	27.90	284,519	594	287	Cyanidin-3-O-rutinoside	0.297 ^c^
14.060						
**Blackcurrant**						
1	9.85	277,525	465	303	Delphinidin-3-O-glucoside	2.75 ^c^
2	10.98	276,526	300	283/252	Delphinidin-3-O-rutinoside	7.89 ^a^
3	13.14	282,517	449	287	Cyanidin-3-O-glucoside	0.78 ^d^
4	14.83	280,518	595	287	Cyanidin-3-O-rutinoside	4.74 ^b^
**16.17**						
**Cranberry**						
1	11.37	279,516	449	287	Cyanidin-3-O-galactoside	14.02 ^a^
2	13.03	280,517	449	287	Cyanidin-3-O-glucoside	0.85 ^c^
3	15.31	278,516	419	287	Cyanidin-3-O-arabinoside	1.80 ^b^
4	26.55	280,528	623	464	Malvidin-3-O-glucoside-4-vinylcathecol	0.25 ^d^
5	28.60	278,526	535	331	Malvidin-6-acetyl-3-galactoside	0.21 ^d^
17.13						
**Raspberry**						
1	10.31		611	287	Cyanidin-3-O-sophoroside	8.99 ^a^
2	13.16		757	611/430/286	Cyanidin-3-O-sophoroside-5-rhamnoside	1.90 ^b^
10.89						

The mean values are obtained from triplicate determinations and expressed on fresh weight (FW) basis. Mean values of concentration, marked with different letters (a–e) in the same column, are significantly different (*p* < 0.05).

**Table 4:** Relative percentage (% of total peaks area) of volatile compounds found in berry jams.

	Compound	t_R_ (min)	Blueberry	Blackberry and Blackcurrant	Blackcurrant	Cranberry	Raspberry	Odor Characteristic Descriptors
	**Alcohols**							
1	1-Butanol 3-methyl	4.58	0.10	0.23	7.14	0.00	0.00	whiskey, malt, burnt
2	1-Butanol 2-methyl	4.676	0.00	0.34	7.06	0.00	0.00	malt
3	Furfuryl alcohol	5.737	0.05	0.00	0.00	0.00	0.00	fermented, creamy, caramel
4	1-Hexanol	12.647	0.23	0.00	0.00	0.00	0.00	flowery, sweet, toasty, green
5	2-Heptanol	14.593	0.00	0.19	0.00	0.00	0.00	herbal, fruity, musty
	Total		0.38	0.76	14.20	0.00	0.00	
	**Aldehydes**							
6	Hexanal	7.343	0.14	0.23	0.80	0.50	0.00	fresh, green, fruity
7	2-Heptanal	17.448	0.00	0.21	1.01	0.00	0.00	green
8	Benzaldehyde	17.53	0.18	0.30	1.55	2.02	0.00	almond, burnt sugar
	**Total**		**0.32**	**0.74**	**3.36**	**2.52**	**0.00**	
	**Esters**							
9	Methyl butanoate	4.094	0.00	0.00	3.27	0.00	0.00	ether, fruit, sweet
10	Methyl 2-methylbutanoate	6.081	0.00	0.00	0.00	0.71	0.00	apple, fruity
11	Ethyl 3-methylbutanoate	11.436	1.11	0.00	0.00	0.00	0.00	sweet, anise, fruity, apple
12	Methyl benzoate	23.219	0.00	0.00	0.00	0.18	0.00	flowery, honey, herbal
	Total		1.11	0.00	3.27	0.89	0.00	
	**Terpene hydrocarbons and oxygenated derivatives**							
13	Linalool oxide	17.97	0.12	0.00	1.24	0.89	0.00	floral, fresh, lemon
14	β-Myrcene	19.029	0.00	0.00	0.00	0.00	0.36	spicy, ethereal
15	∆-3-Carene	19.683	0.27	1.69	28.59	0.00	0.00	citrus fruits, orange peel
16	p-Cymene	20.406	0.50	2.95	14.96	3.19	0.42	citrus
17	D-Limonene	20.592	94.80	89.85	11.40	79.95	98.82	fruity
18	Eucalyptol	20.714	0.29	0.00	1.79	0.93	0.00	minty, pine, sweet
19	trans-β-Ocimene	20.977	0.00	0.00	1.30	0.00	0.00	sweet, herb, citrus
20	γ-Terpinene	21.801	0.00	0.00	1.28	0.00	0.00	citrus, terpeny, sweet, fruity
21	α- Terpinolen	22.864	0.10	0.39	1.22	0.00	0.05	woody, fruity, sweet, piney, anise
22	p-Cymenene	23.045	0.34	1.05	4.41	0.25	0.15	phenolic, spicy, musty, nutty
23	α-Terpineol	27.127	1.09	1.18	2.14	0.85	0.20	anise, mint
24	Caryophyllene	37.916	0.00	0.21	4.54	0.00	0.00	woody, spicy, fruity, sweet
25	α-Caryophyllene	39.649	0.00	0.00	1.19	0.00	0.00	fruity, woody
	Total		97.51	97.32	74.06	86.06	100.00	
	**Others**							
26	2H-Pyran, 3,4-dihydro-6-methyl	4.991	0.08	0.00	0.00	2.90	0.00	
27	Acetophenone		0.37	0.64	2.46	2.19	0.00	sweet, flower, almond
28	Benzoic Acid	26.103	0.23	0.30	1.82	3.32	0.00	winey, balsamic, very weak
29	n.i.	15.959	0.00	0.24	0.00	0.00	0.00	
30	n.i.	30.854	0.00	0.00	0.00	2.12	0.00	
	Total		0.68	1.18	4.28	10.53	0.00	

n.i.-not identified.
